# Time to metastasis as a prognostic factor in metastatic urothelial carcinoma: results from the ARON-2 study

**DOI:** 10.1007/s10585-025-10382-x

**Published:** 2025-11-21

**Authors:** Renate Pichler, Gerald Klinglmair, Kirstin Binz, Enrique Grande, Alina Pirshtuk, Hideki Takeshita, Yüksel Ürün, Javier Molina-Cerrillo, Zin W. Myint, Alfonso Gómez de Liaño, Augusto Mota, Alessia Salfi, Wataru Fukuokaya, Enrico Sammarco, Martin Angel, Jakub Kucharz, Deniz Tural, Ondřej Fiala, Alejo Rodriguez-Vida, Franco Morelli, Alexandr Poprach, Mobin Safi, Alvaro Pinto, Francesco Massari, Sebastiano Buti, Shilpa Gupta, Fernando Sabino Marques Monteiro, Andrey Soares, Nicola Battelli, Ravindran Kanesvaran, Matteo Santoni

**Affiliations:** 1 Department of Urology, Comprehensive Cancer Center Innsbruck, Medical University of Innsbruck, Innsbruck, Austria; 2 Division of Medical Oncology, Department of Internal Medicine, University of Kansas Cancer Center, Overland Park, USA; 3 Department of Medical Oncology, MD Anderson Cancer Center Madrid, Madrid, Spain; 4 Department of Oncology, Second Faculty of Medicine, Charles University and University Hospital Motol, V Uvalu 84, 150 06 Prague, Czech Republic; 5 Department of Urology, Saitama Medical Center, Saitama Medical University, Kawagoe, Saitama Japan; 6 Department of Medical Oncology, Faculty of Medicine, Ankara University, 06620 Ankara, Turkey; 7 Department of Medical Oncology, Hospital Ramón y Cajal, Madrid, Spain; 8 Division of Medical Oncology, Department of Internal Medicine, Markey Cancer Center, University of Kentucky, Lexington, KY USA; 9 Medical Oncology Department, CHU Insular-Materno Infantil, Las Palmas de Gran Canaria, Spain; 10 Clínica AMO - Assistência Multidisciplinar em Oncologia, Salvador, Brazil; 11 Oncology Unit 2, University Hospital of Pisa, 56126 Pisa, Italy; 12 Department of Urology, Jikei University School of Medicine, Tokyo, Japan; 13 Medical Oncology Unit, Livorno Hospital, Azienda Toscana Nord Ovest, 57124 Leghorn, Italy; 14 Clinical Oncology, Genitourinary Oncology Unit, Alexander Fleming Institute, Buenos Aires, Argentina; 15 Department of Uro-Oncology, Maria Sklodowska-Curie National Research Institute of Oncology Warsaw, Warsaw, Poland; 16 Department of Medical Oncology, Koc University Medical Faculty, Istanbul, Turkey; 17 Department of Oncology and Radiotherapeutics, Faculty of Medicine and University Hospital Pilsen, Charles University Prague, Alej Svobody 80, 304 60 Pilsen, Czech Republic; 18 Biomedical Center, Faculty of Medicine in Pilsen, Charles University, Pilsen, Czech Republic; 19 Hospital del Mar, Barcelona, Spain; 20 Medical Oncology Unit, IRCCS Casa Sollievo Della Sofferenza, Foggia, Italy; 21 Masaryk Memorial Cancer Institute, Brno, Czech Republic - Faculty of Medicine, Masaryk University, Brno, Czech Republic; 22 U.O. Oncologia, Ospedale C. Urbani, Jesi, Italy; 23 Servicio de Oncología, Hospital Universitario La Paz, Madrid, Spain; 24 Medical Oncology, IRCCS Azienda Ospedaliero-Universitaria Di Bologna, Bologna, Italy; 25 Department of Medical and Surgical Sciences (DIMEC), University of Bologna, Bologna, Italy; 26 Medical Oncology Unit, University Hospital of Parma, Parma, Italy; 27 Department of Medicine and Surgery, University of Parma, Parma, Italy; 28 Taussig Cancer Institute, Cleveland Clinic, Cleveland, OH USA; 29 Oncology and Hematology Department, Hospital Sírio Libanês, Brasília, Brazil; 30 Latin American Cooperative Oncology Group - LACOG, Porto Alegre, Brazil; 31 Oncology Unit, Hospital Israelita Albert Einstein, São Paulo, SP Brazil; 32 Medical Oncology Unit, Macerata Hospital, Macerata, Italy; 33 Division of Medical Oncology, National Cancer Centre Singapore, Singapore, Singapore

**Keywords:** Chemotherapy, Enfortumab vedotin, Immunotherapy, Time to metastasis, NCT05290038, Pembrolizumab, Urothelial Carcinoma

## Abstract

Metastatic urothelial carcinoma (mUC) may present with metastases at the time of initial diagnosis (synchronous) or develop them during follow-up (metachronous). The impact of the timing of metastasis on the outcome of mUC remains unclear. We aimed to evaluate overall survival (OS) stratified by time to metastasis (TTM) in patients receiving systemic therapy in different lines. Retrospective real-world data from the ARON-2 study were analyzed to compare patient outcomes according to TTM. Cohort 1 included 735 patients receiving first-line platinum-based chemotherapy, Cohort 2 included 1164 patients receiving second-line pembrolizumab, Cohort 3 included 588 patients receiving third-line enfortumab vedotin. TTM (synchronous vs. < 6 months, and ≥ 6 months) significantly influenced overall survival (OS) in Cohort 1 (19.2 vs. 22.3 vs. 27.4 months, *p* = 0.004) and Cohort 2 (14.6 vs. 15.4 vs. 21.2 months, *p* = 0.015), but not in Cohort 3. In the multivariable Cox analysis, TTM remained an independent prognostic parameter of poor OS in Cohort 1 (hazard ratio [HR]: 1.14, 95% confidence interval [CI] 1.02–1.27; *p* = 0.016) and Cohort 2 (HR: 1.12, 95% CI 1.02–1.22; *p* = 0.014). Our findings suggest that the TTM in mUC significantly influences OS in patients receiving first-line platinum-based chemotherapy and second-line pembrolizumab. The prognostic role of TTM should be considered in the future clinical trial designs.

**Graphical abstract:**

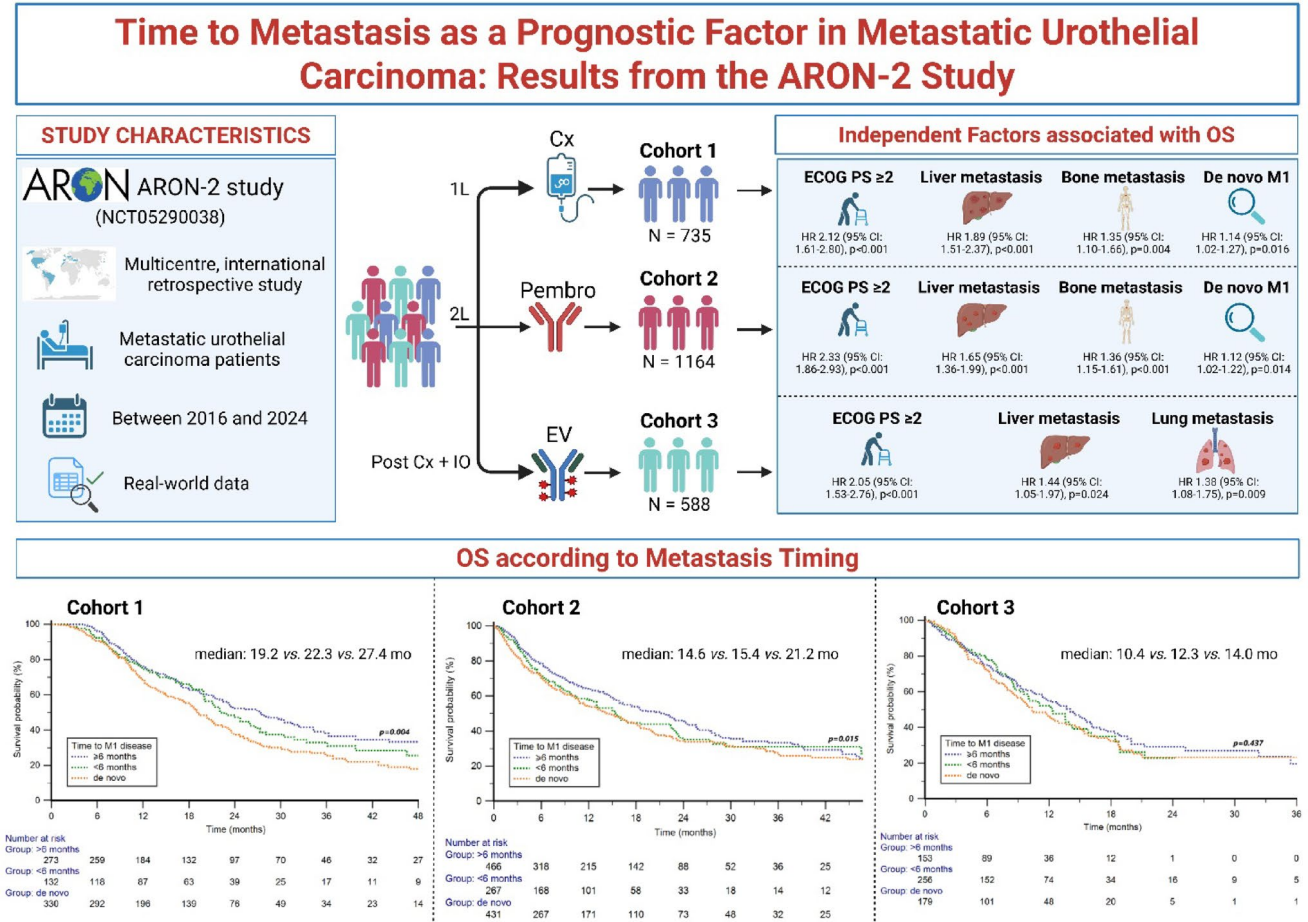

**Supplementary Information:**

The online version contains supplementary material available at 10.1007/s10585-025-10382-x.

## Background

Patients with metastatic urothelial carcinoma (UC) have poor prognosis, with estimated 5-year survival rates of 5–15% [1]. Since pivotal trials published more than 20 years ago established the standard of care for first-line treatment with cisplatin-based combinations showing a median overall survival (OS) of 14–15.2 months (overall response rates of 46% for methotrexate, vinblastine, doxorubicin, and cisplatin (MVAC) and 49% for gemcitabine/cisplatin) [2], the management of metastatic UC (mUC) has mainly stayed the same. This longstanding paradigm was challenged in the last decade by the introduction of immunotherapy using immune checkpoint inhibitors (ICI) [3–6]. In October 2023, the results of two groundbreaking randomised clinical trials (EV-302/KEYNOTE A39 and Checkmate 901) showing a significant OS benefit in the first-line setting against platinum-based chemotherapy were presented [7, 8], revolutionising the therapeutic landscape in the mUC.

The timing of metastatic presentation has been identified as a prognostic factor and a predictor of treatment efficacy in other cancer entities such as prostate cancer and renal cell carcinoma (RCC). In metastatic hormone-sensitive prostate cancer (mHSPC), the median survival of patients with newly diagnosed metastases (synchronous mHSPC) is approximately 50 months with ADT alone. However, this varies significantly due to the heterogeneity of the metastatic patient population [9]. In detail, patients with metachronous metastatic disease (after radical local treament of the primary tumor) versus those with synchronous metastatic disease have been shown to have generally a better prognosis [10]. Moreover, in subgroup analyses from GETUG-AFU 15 and CHAARTED the beneficial effect of the addition of docetaxel to ADT was most evident in men with synchronous metastatic high-volume disease [11, 12]. In metastatic RCC, the time interval from primary diagnosis to metastatic disease (systemic therapy start) is an established key parameter for determining prognosis according to the International Metastatic Renal Cell Carcinoma Database (IMDC) and Memorial Sloan Kettering Cancer Center (MSKCC) risk criteria [13, 14].

However, it remains uncertain whether a similar correlation exists between the timing of metastatic presentation and treatment outcomes in mUC.

The ARON-2 project is a large international real-world registry collecting clinical data from mUC patients treated across multiple countries. Several analyses from this cohort have already been published, addressing different aspects of mUC management and outcomes, including global real-world experiences with pembrolizumab after platinum-based chemotherapy [15], its use in cisplatin-ineligible and upper tract UC patients [16, 17], the impact of concomitant medications [18], sex-related survival differences [19], and the effect of radiotherapy or bone-targeting agents in combination with immunotherapy [20, 21]. Additional work within the ARON-2EV extension has evaluated real-world outcomes with enfortumab vedotin [22, 23]. The present analysis builds upon these previous findings by investigating Time to Metastasis (TTM) as an independent prognostic factor across treatment lines in the ARON-2 population, an aspect not previously explored within this real-world registry.

## Patients and methods

### Study design and population

This study represents a retrospective analysis of clinical data from adult patients (≥ 18 years) diagnosed with mUC, included in the ARON-2 registry (Figs. S1 and S2). The study involved three distinct cohorts: Cohort 1, including patients receiving first-line platinum-based chemotherapy; Cohort 2, including patients who received second-line pembrolizumab; Cohort 3, including patients treated with third-line enfortumab vedotin. As ARON-2 is a longitudinal registry, the cohorts partially overlap; the relationship between the three cohorts is illustrated in Fig. S2.

All analyses were performed independently within each line of therapy.; all analyses were performed independently within each line of therapy.

Data for these cohorts were collected from 80 institutions across various countries, for patients who received either platinum-based chemotherapy, pembrolizumab or enfortumab vedotin between January 1, 2016, and October 31, 2024. Key patient data, including demographic information (age, sex), tumor histology, Eastern Cooperative Oncology Group Performance Status (ECOG-PS), TTM, metastatic sites, prior surgeries, chemotherapy regimens, treatment duration, and response to therapy (assessed using RECIST version 1.1) [24], were included in the analysis. Clinical and histo-pathological informations were obtained from each center’s medical records, as per standard clinical practice, including physical examinations, laboratory tests, and imaging (CT and MRI scans), in line with local institutional guidelines. Patients with incomplete clinical or outcome data were excluded from the study.

### Study objectives

The primary objective was to assess the prognostic significance of TTM in mUC patients, treated with either platinum-based chemotherapy (Cohort 1), pembrolizumab (Cohort 2) or enfortumab vedotin (Cohort 3). Objective responses, categorized as progression disease (PD), stable disease (SD), partial response (PR), or complete response (CR), were evaluated according to RECIST version 1.1. Key outcome measures included overall response rate (ORR) and overall survival (OS). OS was defined as the time from the initiation of platinum-based chemotherapy (Cohort 1), pembrolizumab (Cohort 2) or enfortumab vedotin (Cohort 3) until death from any cause. ORR was determined by summing CR and PR responses.

### Statistical analysis

To compare OS between groups, the Kaplan–Meier method was used, with differences assessed by the log-rank test. Patients alive at the last follow-up were censored. The median follow-up duration was also estimated with the Kaplan–Meier method. Cox proportional hazards models were applied to assess the multivariable impact on survival and to calculate hazard ratios (HRs) with 95% confidence intervals (CIs). Fisher’s exact test was utilized for pairwise comparisons of categorical variables, while chi-square tests were applied for multiple categorical comparisons. Univariable and multivariable analyses were performed by using Cox proportional hazard models, Hazard Ratio (HR) and their 95% confidence intervals (CI). Landmark analysis was performed designating 6 months as the time point during follow-up period in order to reduce potential biases related to the follow-up time. A *p*-value < 0.05 was considered statistically significant.

## Results

### Study population

The overall study population was composed of three cohorts. Cohort 1 included 735 patients treated with first-line platinum-based chemotherapy, while patients treated with adjuvant or neoadjuvant chemotherapy followed by pembrolizumab and those receiving avelumab switch maintenance following first-line chemotherapy were not included due to the main objective of the analysis. Cohort 2 included 1164 patients treated with second-line pembrolizumab. Cohort 3 included 588 patients receiving third-line enfortumab vedotin (EV) (Figs. S1 and S2). Overview of patients' baseline characteristics is summarized in Table 1. The number of patients and the overlap of the three cohorts is illustrated in Fig. S3.

**Table 1:** Descriptive characteristics of the three cohorts based on the time to metastasis (TTM)

Characteristics	Platinum-based chemotherapy (Cohort 1)				Pembrolizumab (Cohort 2)				Enfortumab vedotin (Cohort 3)			
	Synchronous metastatic disease	TTM < 6 months	TTM ≥ 6 months	*p*-value	Synchronous metastatic disease	TTM < 6 months	TTM ≥ 6 months	*p*-value	Synchronous metastatic disease	TTM within < 6 months	TTM ≥ 6 months	*p*-value
Total patients	330 (100)	132 (100)	273 (100)	–	431 (100)	267 (100)	466 (100)	–	179 (100)	256 (100)	153 (100)	–
*Sex*												
Male	231 (70)	93 (70)	208 (76)	0.551	299 (69)	196 (73)	358 (77)	0.444	137 (77)	189 (74)	123 (80)	0.602
Female	99 (30)	39 (30)	65 (24)		132 (31)	71 (27)	108 (23)		42 (23)	67 (26)	30 (20)	
Age ≥ 70 y	159 (48)	78 (59)	167 (61)	0.137	230 (53)	137 (51)	237 (51)	0.948	94 (53)	119 (46)	85 (56)	0.348
*ECOG performance status*												
0–1	287 (87)	118 (89)	250 (92)	0.515	369 (86)	234 (88)	423 (91)	0.541	145 (81)	223 (87)	125 (82)	0.125
≥ 2	43 (13)	14 (11)	23 (8)		62 (14)	33 (12)	43 (9)		34 (19)	33 (13)	28 (18)	
*Tumor histology*												
Pure urothelial carcinoma	248 (75)	102 (77)	221 (81)	0.584	318 (74)	210 (79)	377 (81)	0.469	125 (70)	189 (74)	110 (72)	0.820
Minor or mixed variants	82 (25)	30 (23)	52 (19)		113 (26)	57 (21)	89 (19)		54 (30)	67 (26)	43 (28)	
*Primary tumor location*												
Upper urinary tract	103 (31)	42 (32)	89 (33)	0.955	121 (28)	72 (27)	140 (30)	0.891	126 (70)	182 (71)	110 (72)	0.953
Lower urinary tract	227 (69)	90 (68)	184 (67)		310 (72)	195 (73)	326 (70)		53 (30)	74 (29)	43 (28)	
*Common sites of metastasis*												
Lymph nodes (non-regional)	240 (73)	86 (65)	162 (59)	0.112	312 (72)	178 (67)	282 (61)	0.255	135 (75)	143 (56)	91 (59)	**0.011**
Lung	143 (43)	49 (37)	98 (36)	0.546	169 (39)	91 (34)	158 (34)	0.696	63 (35)	88 (34)	66 (43)	0.353
Bone	107 (32)	38 (29)	77 (28)	0.813	134 (31)	73 (27)	133 (29)	0.823	45 (25)	55 (21)	32 (21)	0.735
Liver	83 (25)	23 (17)	41 (15)	0.162	93 (22)	47 (18)	76 (16)	0.541	36 (20)	31 (12)	24 (16)	0.304

ECOG-PS = Eastern cooperative oncology group-performance status; TTM = time to metastasis; values in bold are statistically significant.

### Outcomes with first-line platinum-based chemotherapy (Cohort 1)

#### Survival analysis

**Fig. 1:**
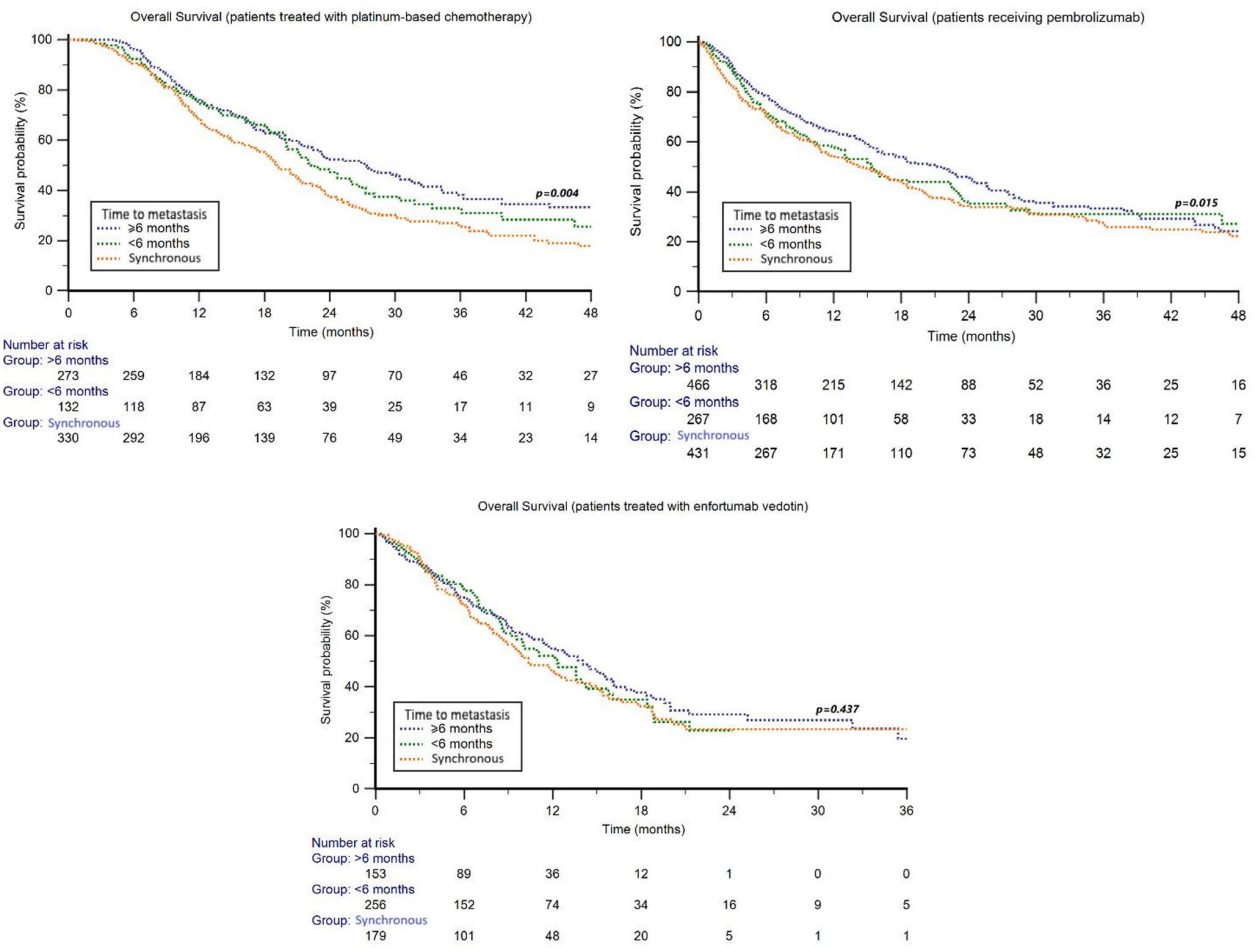
Overall survival in patients treated with platinum-based chemotherapy (Cohort 1), pembrolizumab (Cohort 2) or enfortumab vedotin (Cohort 3) stratified by time to metastasis (TTM)

The median follow-up was 31.3 months (95% CI 28.9–34.1). The median OS in the Cohort 1 was 22.0 (95% CI 20.3–116.5) months. Stratified by TTM, the median OS was 19.2 months (95%CI 17.6–21.3) in patients with synchronous metastaticdisease, 22.3 months (95% CI 19.9–116.5) in those with TTM < 6 months and 27.4 months (95% CI 22.4–31.8) in those with TTM ≥ 6 months (*p* = 0.004) (Fig. 1). In addition, 6-month landmark analysis confirmed significant differences in OS, with synchronous metastatic disease presenting the poorest OS compared with metachronous disease with TTM < 6 months and TTM ≥ 6 months (median 18.8 vs. 22.3, and 23.7 months, *p* = 0.049) (Fig. S3).

**Fig. 2:**
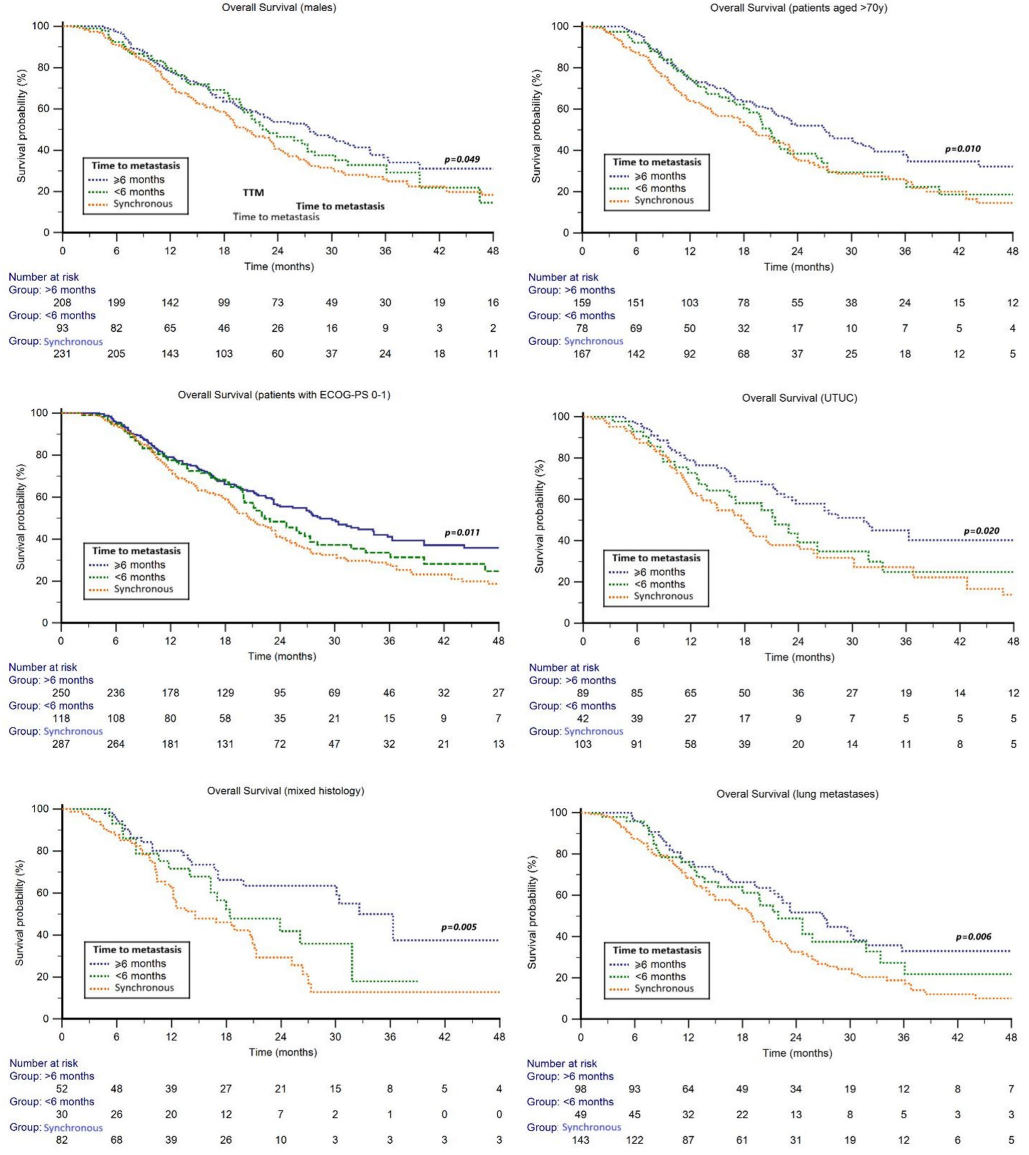
Overall survival in patients treated with first-line platinum-based chemotherapy stratified by time to metastasis (TTM) and clinic-pathological features

Results of subgroup analyses showed significant differences in OS according to TTM in males, patients aged ≥ 70 years, those with ECOG-PS 0–1, upper tract tumors, mixed histology, and in patients with lung metastases. Across all these subgroups, patients with synchronous metastatic disease had the poorest outcomes, while the longest OS was observed in those with TTM ≥ 6 months (*p* = 0.049, 0.010, 0.011, 0.020, 0.005, and 0.006, respectively) (Fig. 2). The results of subgroup analyses are presented in detail in Table S1. The results of subgroup analyses are presented in detail in Table S1. Univariable and multivariable analyses are summarized in Table 2. The negative prognostic impact TTM on OS was maintained at multivariate Cox analysis (HR = 1.14; 95% CI: 1.02–1.27; *p* = 0.016).

**Table 2:** Univariable and multivariable analyses in patients receiving first-line platinum-based chemotherapy (Cohort 1), second-line pembrolizumab (Cohort 2), and third-line enfortumab vedotin (Cohort 3)

Overall survival (overall population)	Platinum-based chemotherapy (Cohort 1)				Pembrolizumab (Cohort 2)				Enfortumab vedotin (Cohort 3)			
	Univariable cox regression		Multivariable cox regression		Univariable cox regression		Multivariable cox regression		Univariable cox regression		Multivariable cox regression	
	HR (95% CI)	*p*-value	HR (95% CI)	*p*-value	HR (95% CI)	*p*-value	HR (95% CI)	*p*-value	HR (95% CI)	*p*-value	HR (95% CI)	*p*-value
Sex (females vs. males)	1.14 (0.93–1.40)	0.212			1.13 (0.95–1.34)	0.179			1.07 (0.81–1.41)	0.630		
Age ≥ 70 y (Y vs. N)	1.15 (0.95–1.39)	0.154			1.09 (0.93–1.28)	0.289			0.86 (0.68–1.09)	0.210		
ECOG PS (≥ 2 vs. 0–1)	2.34 (1.77–3.08)	**< 0.001**	2.12 (1.61–2.80)	**< 0.001**	2.59 (2.07–3.24)	**< 0.001**	2.33 (1.86–2.93)	**< 0.001**	2.29 (1.72–3.04)	**< 0.001**	2.05 (1.53–2.76)	**< 0.001**
Histology (pure UC vs. variants)	0.87 (0.69–1.10)	0.257			0.97 (0.80–1.18)	0.786			0.83 (0.64–1.08)	0.167		
Upper versus lower urinary tract	1.01 (0.82–1.23)	0.934			0.98 (0.82–1.16)	0.800			0.80 (0.61–1.05)	0.107		
TTM (synchronous vs. < 6 months vs. ≥ 6 months)	1.19 (1.07–1.32)	**0.001**	1.14 (1.02–1.27)	**0.016**	1.14 (1.04–1.25)	**0.004**	1.12 (1.02–1.22)	**0.014**	1.05 (0.90–1.24)	0.515		
Lymph node (Y vs. N)	0.95 (0.78–1.16)	0.597			0.85 (0.72–1.00)	0.051			1.01 (0.78–1.30)	0.950		
Lung metastases (Y vs. N)	1.20 (0.99–1.45)	0.062			1.22 (1.04–1.44)	**0.016**	1.16 (0.98–1.37)	0.078	1.41 (1.11–1.80)	**0.005**	1.38 (1.08–1.75)	**0.009**
Bone metastases (Y vs. N)	1.43 (1.17–1.75)	**< 0.001**	1.35 (1.10–1.66)	**0.004**	1.46 (1.24–1.73)	**< 0.001**	1.36 (1.15–1.61)	**< 0.001**	1.01 (0.77–1.34)	0.919		
Liver metastases (Y vs. N)	1.98 (1.59–2.48)	**< 0.001**	1.89 (1.51–2.37)	**< 0.001**	1.72 (1.42–2.08)	**< 0.001**	1.65 (1.36–1.99)	**< 0.001**	1.65 (1.21–2.24)	**0.002**	1.44 (1.05–1.97)	**0.024**

ECOG-PS = Eastern cooperative oncology group-performance status; Y = yes; N = no; TTM = time to metastasis; values in bold are statistically significant.

#### Objective response

The overall response to therapy in Cohort 1 was 6% CR, 22% PR, 25% SD, and 47% PD, corresponding to an ORR of 28%. In patients with synchronous metastatic disease, response rates were 5% CR, 31% PR, 21% SD, and 42% PD (ORR = 36%). In those with TTM < 6 months, the rates were 5% CR, 29% PR, 28% SD, and 38% PD (ORR = 34%), while patients with TTM ≥ 6 months showed 8% CR, 30% PR, 27% SD, and 35% PD (ORR = 38%). Differences among the three groups were not statistically significant in terms of ORR (*p* = 0.841) or primary refractory disease rate (*p* = 0.594).

### Outcomes with second-line pembrolizumab (Cohort 2)

#### Survival analysis

The median follow-up was 20.6 months (95% CI 19.0–22.3). The median OS in Cohort 2 was 16.5 months (95% CI 15.2–111.2). Stratified by TTM, the median OS was 14.6 months (95% CI 11.5–111.2) in patients with synchronous metastatic disease, 15.4 months (95% CI 13.0–86.9) in those with TTM < 6 months and 21.2 months (95%CI 16.4–91.0) in those with TTM ≥ 6 months (*p* = 0.015) (Fig. 1). The 6-month landmark analysis confirmed significant differences in OS, with synchronous metastatic disease presenting the poorest OS compared with metachronous M1 disease with TTM < 6 months and TTM ≥ 6 months (median OS 15.5 vs. 16.8, and 24.3 months, *p* = 0.023) (Fig. S3).

**Fig. 3:**
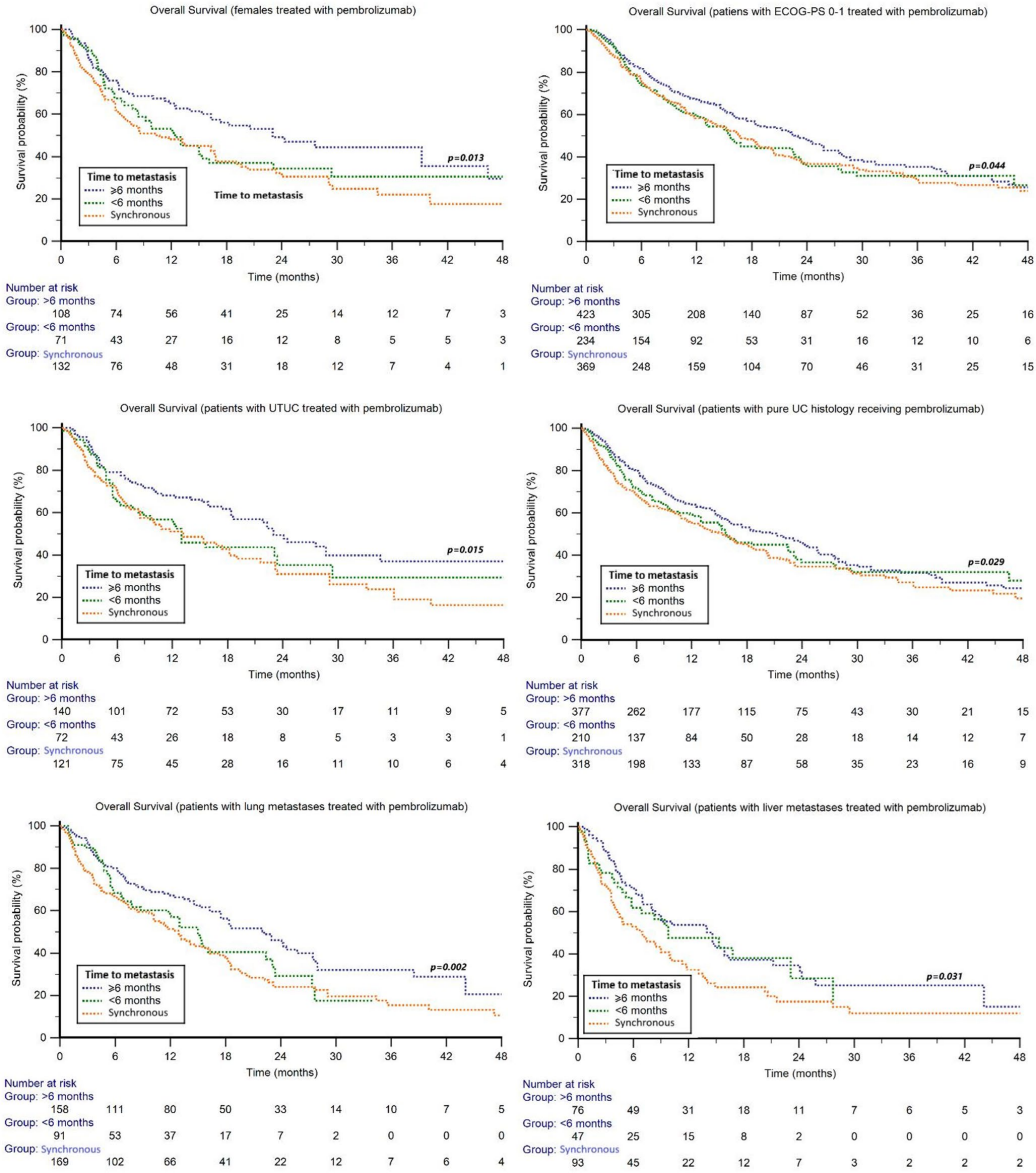
Overall survival in patients treated with second-line pembrolizumab stratified by time to metastasis (TTM) and clinic-pathological features

Results of subgroup analyses showed significant differences in OS according to TTM in females, patients with ECOG-PS 0–1, upper tract tumors, pure UC histology, and in those with lung or liver metastases. Across all these subgroups, the poorest outcomes were observed in patients with synchronous metastatic disease, whereas patients with TTM ≥ 6 months consistently achieved the longest survival (*p* = 0.013, 0.044, 0.015, 0.029, 0.002, and 0.031, respectively) (Fig. 3). The results of subgroup analyses are presented in detail in Table S1.

The prognostic impact of TTM remained an independent prognostic factor for OS in the multivariable Cox analysis (HR = 1.12; 95% CI, 1.02–1.22; *p* = 0.014).

#### Objective response

The overall response in Cohort 2 was 8% CR, 22% PR, 23% SD, and 47% PD, corresponding to an ORR of 31%. In patients with synchronous metastatic disease, response rates were 5% CR, 18% PR, 20% SD, and 57% PD (ORR = 23%). In those with TTM < 6 months, the rates were 9% CR, 21% PR, 20% SD, and 50% PD (ORR = 30%), while patients with TTM ≥ 6 months showed 9% CR, 26% PR, 27% SD, and 38% PD (ORR = 35%). Differences among the three groups were not statistically significant in terms of ORR (*p* = 0.173), whereas the rate of primary refractory disease differed significantly (*p* = 0.025).

### Enfortumab vedotin (Cohort 3)

#### Survival analysis

The median follow-up was 13.2 months (95% CI 10.6–52.9). The median OS in the Cohort 3 was 12.4 months (95% CI 10.7–14.1). Stratified by TTM, the median OS was 10.4 months (95%CI 8.5–47.3) in patients with synchronous metastatic disease, 12.3 months (95% CI 9.6–14.3) in those with TTM < 6 months and 14.0 months (95% CI 11.4–16.0) in those with TTM ≥ 6 months (*p* = 0.437) (Fig. 1).

**Fig. 4:**
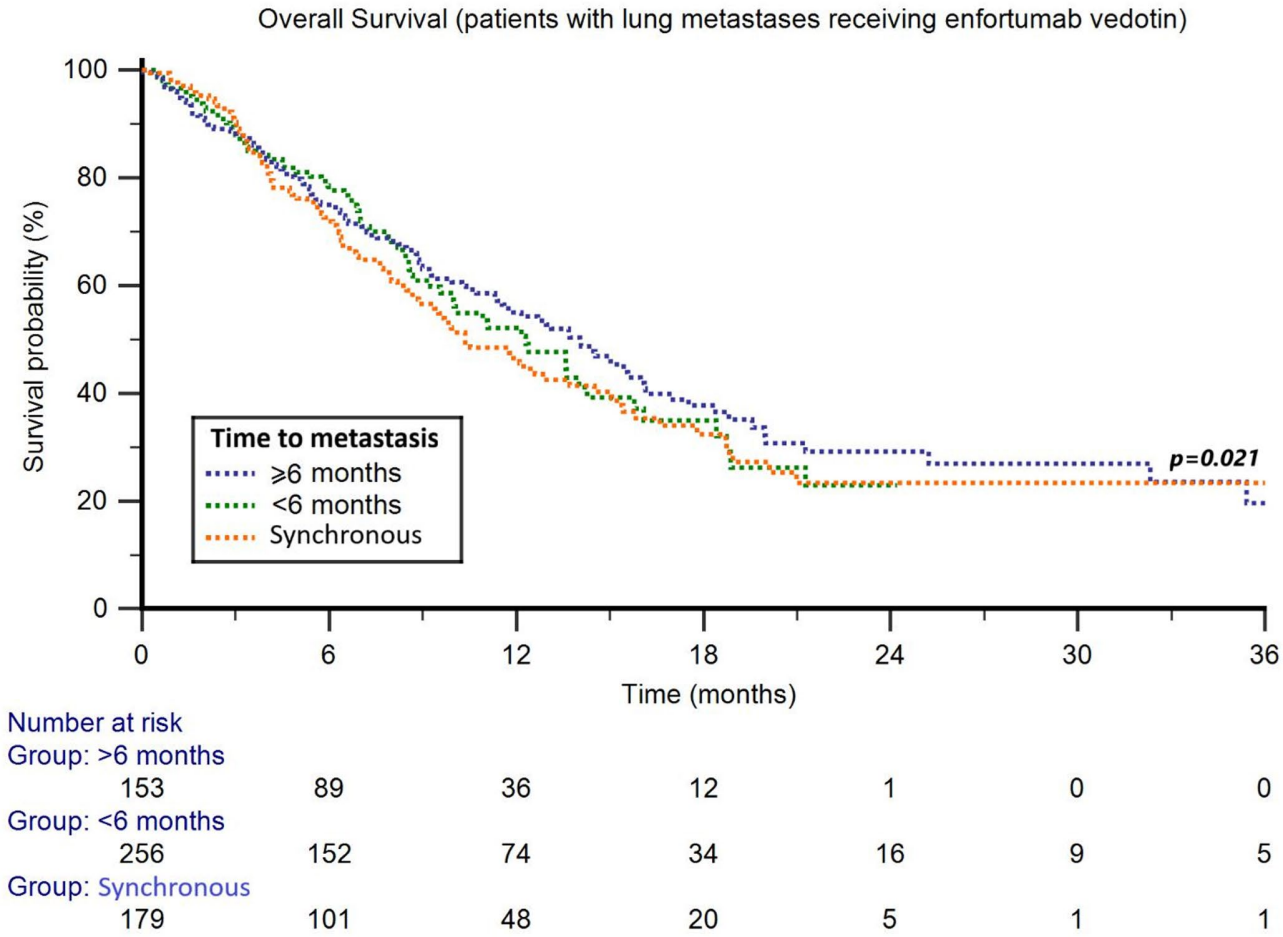
Overall survival in patients with lung metastases treated with third-line enfortumab vedotin stratified by time to metastasis (TTM)

Focusing on subgroups, significant differences according to TTM were observed only in patients with lung metastases, with median OS of 6.4 months for synchronous metastatic disease, 10.7 months for patients with TTM < 6 months, and 13.6 months for those with TTM ≥ 6 months (*p* = 0.021) (Fig. 4). Univariable and multivariable Cox analyses of OS are summarized in Table 2.

#### Objective response

The overall response to therapy in Cohort 3 was 9% CR, 37% PR, 26% SD, and 28% PD, corresponding to an ORR of 46%. In patients with synchronous metastatic disease, response rates were 12% CR, 35% PR, 23% SD, and 30% PD (ORR = 47%). In those with TTM < 6 months, the rates were 8% CR, 36% PR, 27% SD, and 29% PD (ORR = 44%), while patients with TTM ≥ 6 months showed 5% CR, 39% PR, 29% SD, and 27% PD (ORR = 44%). Differences among the three groups were not statistically significant in terms of ORR (*p* = 0.886).

## Discussion

The impact of TTM on mUC patient outcome is poorly characterized. For the first time, this study reports its influence on mUC prognosis in one of the largest real-world patient populations with synchronous metastatic disease being an independent prognostic factor of poor OS undergoing platinum-based first-line chemotherapy as well as second-line pembrolizumab.

In the chemotherapy era, the Bajorin risk factors and Bellmunt risk score were widely accepted benchmarks for stratifying mUC patients and predicting survival [25, 26]. The Bellmunt risk score stratifies patients into four risk groups based on three prognostic factors: ECOG-PS > 0, hemoglobin (HGB) levels < 10 g/dL, and presence of liver metastases [26]. With the introduction of immunotherapy, the „enhanced CRP-Bellmunt risk score“ was developed using data from the IMvigor210 (development) and IMvigor211 (external validation) trials [27], aiming to improve (i) patient selection in clinical trials with immunotherapy and (ii) prognostic informations for mUC patients undergoing immunotherapy. In contrast to mUC, TTM has already been an established prognostic parameter for OS in several types of cancer. In oligometastatic NSCLC, a meta-analysis has also shown that OS is inferior in patients with synchronous metastatic disease compared to those with metachronous disease [28]. Moreover, TTM has been included in various prognostic risk scores regarding mRCC [13, 14]. The factor "time from diagnosis to first-line systemic treatment < 1 year" represents an established prognostic parameter for predicting survival in mRCC [12, 13]. Additionally, mRCC patients with synchronous disease compared to those with metachronous disease showed also more adverse prognostic features, significantly shorter time to treatment failure, and poor survival outcomes [29].

In our study, patients with synchronous metastatic disease showed significantly poorest OS with both first-line platinum-based chemotherapy and second-line immunotherapy with pembrolizumab compared to those who developed metastases after local therapy of the primary tumor. Landmark analyses at 6 month after systemic therapy initiation were performed to minimize lead-time bias, still confirming significant poor OS in synchronous metastatic patients in comparison to those with metachronous disease. Regarding the first-line chemotherapy, OS benefit was confirmed in metachronous M1 patients (with TTF ≥ 6 months) who were male (*p* = 0.049), older than 70 years (*p* = 0.010), had ECOG-PS 0–1 (*p* = 0.011), upper tract urothelial carcinoma (UTUC) (*p* = 0.02), mixed histology (*p* = 0.005), and lung metastases (*p* = 0.006). Overall, synchronous versus metachronous metastatic disease by intervals TTM < 6 months, and TTM ≥ 6 months correlated with poor OS (median 19.2 vs. 22.3, and 27.4 months, *p* = 0.004).

Focusing on pembrolizumab in the second-line, subgroups of metachronous M1 patients such as women (*p* = 0.013), ECOG-PS 0–1 (*p* = 0.044), UTUC (*p* = 0.015), pure UC histology (*p* = 0.029), lung metastases (*p* = 0.002), and liver metastases (*p* = 0.031) showed the best OS compared to synchronous metastatic disease. In multivariate Cox analysis, synchronous metastatic disease remained an independent prognostic parameter for poor OS (HR = 1.12; 95% CI 1.02–1.22; *p* = 0.014). Interestingly, TTM no longer seems to have an impact on OS in more advanced lines of therapy. In our Cohort 3, including patients who received EV after progression following chemotherapy and pembrolizumab, there was no overall OS difference between the three groups based on TTM, which may be related not only to the effectiveness of EV in this setting but also to a potential selection effect, as patients with synchronous metastatic disease and poorer prognosis were less likely to reach third-line therapy. TTM did not affect the objective response to first-line platinum-based chemotherapy, second–line pembrolizumab, or third-line EV monotherapy. In the literature, only a single study has currently examined the prognostic role of TTM in mUC. Yoshida et al*.* showed that the timing of metastasis influenced prognosis, with synchronous metastases being associated with worse outcomes compared to metachronous metastases in oligometastatic mUC with visceral metastases [30].

Limitations of this study include the retrospective design and multicentre nature with a lack of independent radiological central review. In addition, excluding patients treated with adjuvant or neoadjuvant platinum-based chemotherapy and those with avelumab maintenance may represent a confounding factor. Furthermore, there are limited implications for the clinical practice in view of the evolved new combination treatment landscape in the setting of mUC. Post-hoc exploratory analyses of the EV-302 and CM-901 would be helpful to evaluate the prognostic value of metastasis timing also under EV/Pembrolizumab and Gem-Cis/Nivolumab [7, 8]. Nevertheless, initial timing of metastatic presentation may be worth accounting for in the future mUC clinical trials to explore its prognostic value further. A further limitation that needs to be mentioned is the fact that the exact impact of local therapy of the primary tumor on survival cannot be assessed presenting a potential bias, as the synchronous metastatic disease group did not receive local therapy, unlike the metachronous metastatic disease group.

## Conclusions

This study represents the most extensive analysis to date, demonstrating that TTM influences the survival outcomes of first-line and second-line immunotherapy in mUC but not of the third-line therapy with EV. Patients with synchronous metastatic disease exhibit the poorest OS compared to those with metachronous disease. These findings may aid in patient counseling and should be considered in future clinical trial designs to prevent imbalances between treatment arms.

## Supplementary Information

Below is the link to the electronic supplementary material.

Supplementary Material 1

## Data Availability

The datasets generated and/or analyzed during the current study are not publicly available due to patient data security but are available from the corresponding author on reasonable request.

## Code availability

Not applicable' for that section.
