# Supplementary material for: Time to metastasis as a prognostic factor in metastatic urothelial carcinoma: results from the ARON-2 study
Source: Clin Exp Metastasis. 2025 Nov 21;43(1):1. doi: 10.1007/s10585-025-10382-x (PMC13242420; doi:10.1007/s10585-025-10382-x)
Supplement: Supplementary file 1 — Supplementary Material 1 [file 10585_2025_10382_MOESM1_ESM.docx]

**Supplementary Materials**

**Time to Metastasis as a Prognostic Factor in Metastatic Urothelial Carcinoma: Results from the ARON-2 Study**

Renate Pichler^1^, Gerald Klinglmair^1^, Kirstin Binz^2^, Enrique Grande^3^, Alina Pirshtuk^4^, Hideki Takeshita^5^, Yüksel Ürün^6^, Javier Molina-Cerrillo^7^, Zin W. Myint^8^, Alfonso Gómez de Liaño^9^, Augusto Mota^10^, Alessia Salfi^11^, Wataru Fukuokaya^12^, Enrico Sammarco^13^, Martin Angel^14^, Jakub Kucharz^15^, Deniz Tural^16^, Ondřej Fiala^17,18*^, Alejo Rodriguez-Vida^19^, Franco Morelli^20^, Alexandr Poprach^21^, Mobin Safi^22^, Alvaro Pinto^23^, Francesco Massari^24,25^, Sebastiano Buti^26,27^, Shilpa Gupta^28^, Fernando Sabino Marques Monteiro^29,30^, Andrey Soares^30,31^, Nicola Battelli^32^, Ravindran Kanesvaran^33^, Matteo Santoni^32^

^1^Department of Urology, Comprehensive Cancer Center Innsbruck, Medical University of Innsbruck, Innsbruck, Austria; ^2^Division of Medical Oncology, Department of Internal Medicine, University of Kansas Cancer Center, US; ^3^Department of Medical Oncology, MD Anderson Cancer Center Madrid, Madrid, Spain; ^4^Department of Oncology, Second Faculty of Medicine, Charles University and University Hospital Motol, V Uvalu 84, 150 06, Prague, Czech Republic; ^5^Department of Urology, Saitama Medical Center, Saitama Medical University, Kawagoe, Saitama, Japan; ^6^Department of Medical Oncology, Ankara University Faculty of Medicine, 06620 Ankara, Türkiye; ^7^Department of Medical Oncology, Hospital Ramón y Cajal, Madrid, Spain; ^8^Division of Medical Oncology, Department of Internal Medicine, Markey Cancer Center, University of Kentucky, Lexington, Kentucky, USA; ^9^Medical Oncology Department, CHU Insular-Materno Infantil, Las Palmas de Gran Canaria, Spain; ^10^Clínica AMO - Assistência Multidisciplinar em Oncologia, Salvador, Brazil; ^11^Oncology Unit 2, University Hospital of Pisa, Pisa 56126, Italy; ^12^Department of Urology, Jikei University School of Medicine, Tokyo, Japan; ^13^Medical Oncology Unit, Livorno Hospital, Azienda Toscana Nord Ovest, 57124 Livorno, Italy; ^14^Clinical Oncology, Genitourinary Oncology Unit, Alexander Fleming Institute, Buenos Aires, Argentina; ^15^Department of Uro-Oncology, Maria Sklodowska-Curie National Research Institute of Oncology Warsaw, Warsaw, Poland; ^16^Department of Medical Oncology, Koc University Medical Faculty, Istanbul, Türkiye; ^17^Department of Oncology and Radiotherapeutics, Faculty of Medicine and University Hospital in Pilsen, Charles University, Pilsen, Czech Republic; ^18^Biomedical Center, Faculty of Medicine in Pilsen, Charles University, Pilsen, Czech Republic; ^19^Hospital del Mar, Barcelona, Spain; ^20^Medical Oncology Unit, IRCCS Casa Sollievo della Sofferenza, Foggia, Italy; ^21^Masaryk Memorial Cancer Institute, Brno, Czech Republic - Faculty of Medicine, Masaryk University, Brno, Czech Republic; ^22^U.O. Oncologia, Ospedale C. Urbani, Jesi, Italy; ^23^Servicio de Oncología, Hospital Universitario La Paz, Madrid, Spain; ^24^Medical Oncology, IRCCS Azienda Ospedaliero-Universitaria di Bologna, Bologna, Italy; ^25^Department of Medical and Surgical Sciences (DIMEC), University of Bologna, Bologna, Italy; ^26^Medical Oncology Unit, University Hospital of Parma, Parma, Italy; ^27^Department of Medicine and Surgery, University of Parma, Parma, Italy; ^28^Taussig Cancer Institute, Cleveland Clinic, Cleveland, OH, USA; ^29^Oncology and Hematology Department, Hospital Sírio Libanês, Brasília, Brazil; ^30^Latin American Cooperative Oncology Group - LACOG, Porto Alegre, Brazil; ^31^Oncology Unit, Hospital Israelita Albert Einstein, São Paulo, SP, Brazil; ^32^Medical Oncology Unit, Macerata Hospital, Macerata, Italy; ^33^Division of Medical Oncology, National Cancer Centre Singapore, Singapore, Singapore

***^§^Correspondence to:** Ondrej Fiala, M.D., Ph.D., Department of Oncology and Radiotherapy, Faculty of Medicine and University Hospital Pilsen, Charles University Prague, alej Svobody 80, CZ-304 60 Pilsen, Czech Republic. Tel.: +42 0728655488, e-mail: [fialao@fnplzen.cz](mailto:fialao@fnplzen.cz)

## **First-line platinum-based chemotherapy (Cohort 1)**

| Subgroup | Synchronous | TTM <6 months | TTM ≥6 months | p-value |
| --- | --- | --- | --- | --- |
| Males | 20.4 (95% CI 18.3–23.5) | 22.9 (95% CI 19.9–116.5) | 27.5 (95% CI 22.0–32.6) | 0.049 |
| Age ≥70y | 18.9 (95% CI 14.6–82.0) | 21.0 (95% CI 17.0–116.5) | 26.9 (95% CI 21.2–32.2) | 0.010 |
| ECOG-PS 0–1 | 20.6 (95% CI 18.7–23.5) | 22.3 (95% CI 20.0–116.5) | 28.4 (95% CI 23.3–34.3) | 0.011 |
| UTUC | 17.6 (95% CI 13.4–77.5) | 21.4 (95% CI 13.8–31.8) | 31.3 (95% CI 22.4–49.0) | 0.020 |
| Mixed histology | 14.6 (95% CI 12.2–82.0) | 18.4 (95% CI 14.0–31.8) | 36.3 (95% CI 19.9–52.2) | 0.005 |
| Lung metastases | 19.1 (95% CI 15.0–21.0) | 22.0 (95% CI 15.3–116.5) | 27.2 (95% CI 21.6–30.4) | 0.006 |

## **Second-line pembrolizumab (Cohort 2)**

| Subgroup | Synchronous | TTM <6 months | TTM ≥6 months | p-value |
| --- | --- | --- | --- | --- |
| Females | 10.7 (95% CI 6.6–60.0) | 12.4 (95% CI 7.8–16.1) | 23.0 (95% CI 15.3–39.2) | 0.013 |
| ECOG-PS 0–1 | 15.6 (95% CI 13.0–111.2) | 16.7 (95% CI 13.8–86.9) | 23.0 (95% CI 18.4–91.0) | 0.044 |
| UTUC | 13.2 (95% CI 8.5–72.4) | 13.0 (95% CI 7.7–23.4) | 23.0 (95% CI 18.4–34.6) | 0.015 |
| Pure UC histology | 15.4 (95% CI 11.7–83.6) | 15.8 (95% CI 13.0–86.9) | 21.2 (95% CI 16.3–91.0) | 0.029 |
| Lung metastases | 12.9 (95% CI 10.0–83.6) | 15.0 (95% CI 8.8–22.4) | 22.2 (95% CI 17.4–25.8) | 0.002 |
| Liver metastases | 7.0 (95% CI 4.3–72.4) | 9.8 (95% CI 5.8–23.1) | 14.6 (95% CI 8.0–16.4) | 0.031 |

## **Third-line enfortumab vedotin (Cohort 3)**

| Subgroup | Synchronous | TTM <6 months | TTM ≥6 months | p-value |
| --- | --- | --- | --- | --- |
| Lung metastases | 6.4 (95% CI 5.0–9.7) | 10.7 (95% CI 8.5–13.7) | 13.6 (95% CI 9.2–18.7) | 0.021 |

ECOG-PS = Eastern Cooperative Oncology Group-Performance Status; UC = urothelial carcinoma; UTUC = upper tract urothelial carcinoma; TTM = Time to Metastasis

**Table S1**. Median overall survival (OS) by subgroups according to time to metastasis (TTM)

**
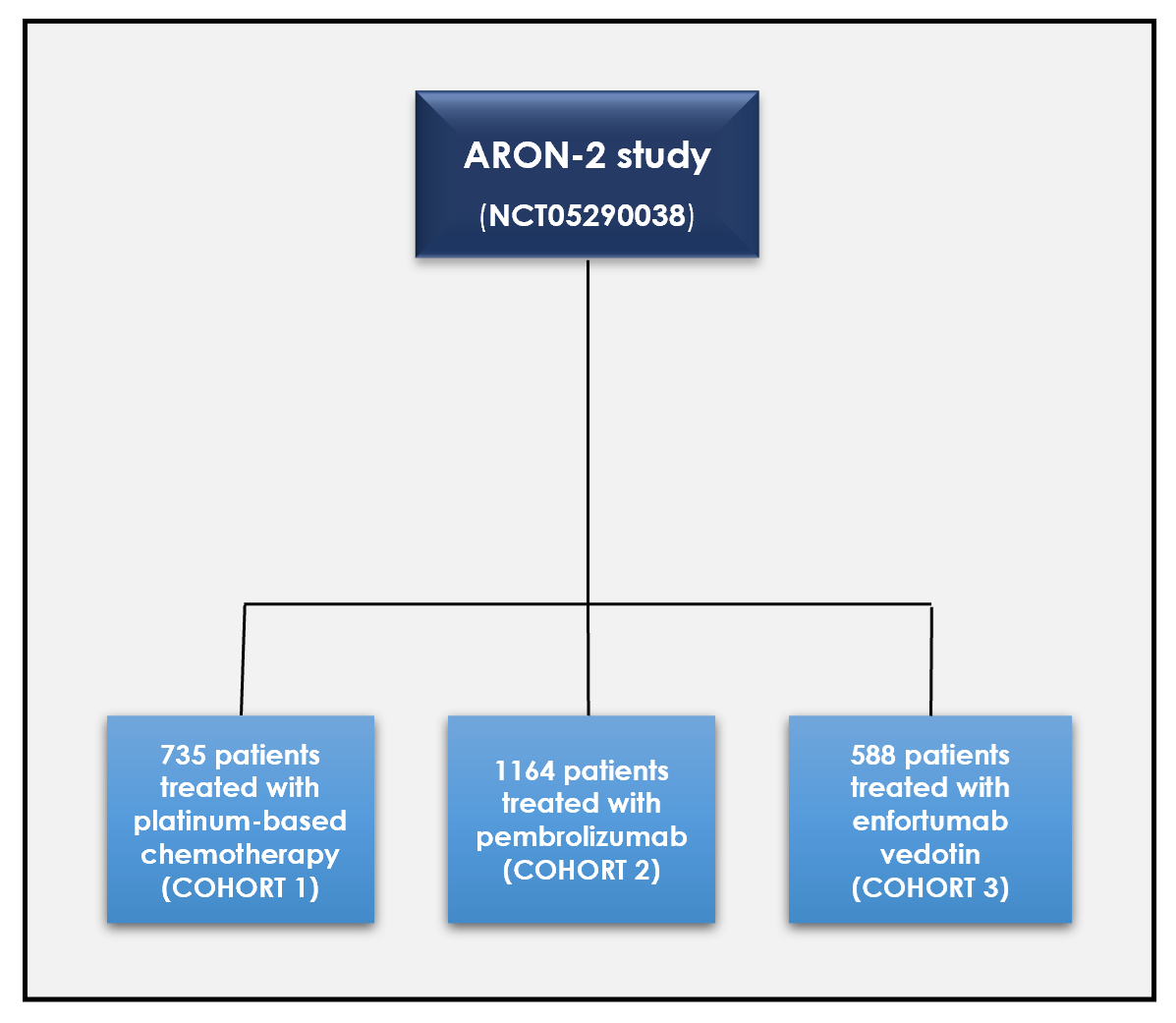
**

**Figure S1.** Selection process from the ARON-2 dataset.

**
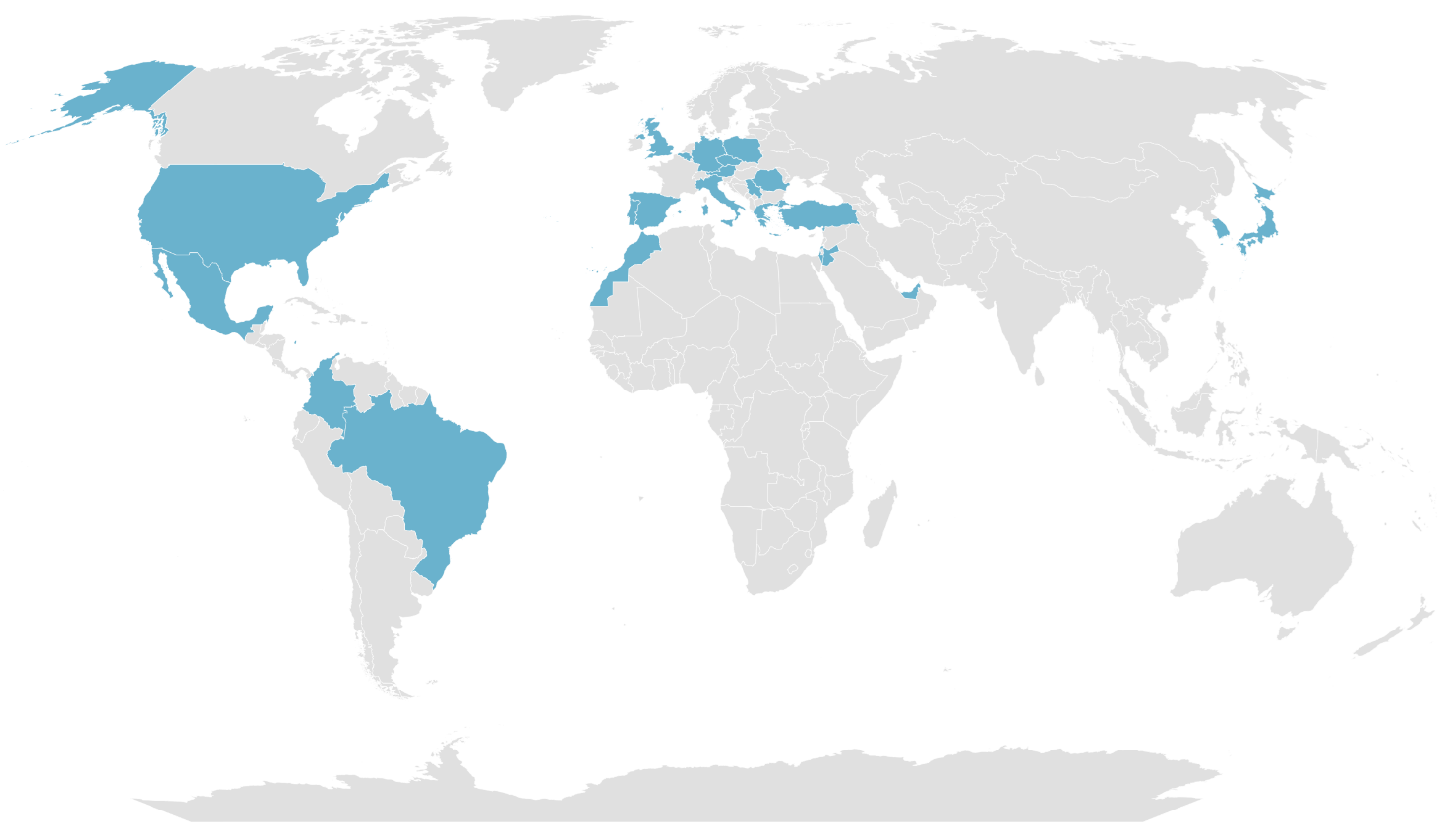
**

**Figure S2.** Map of Countries participating to the ARON-2 Study.

**
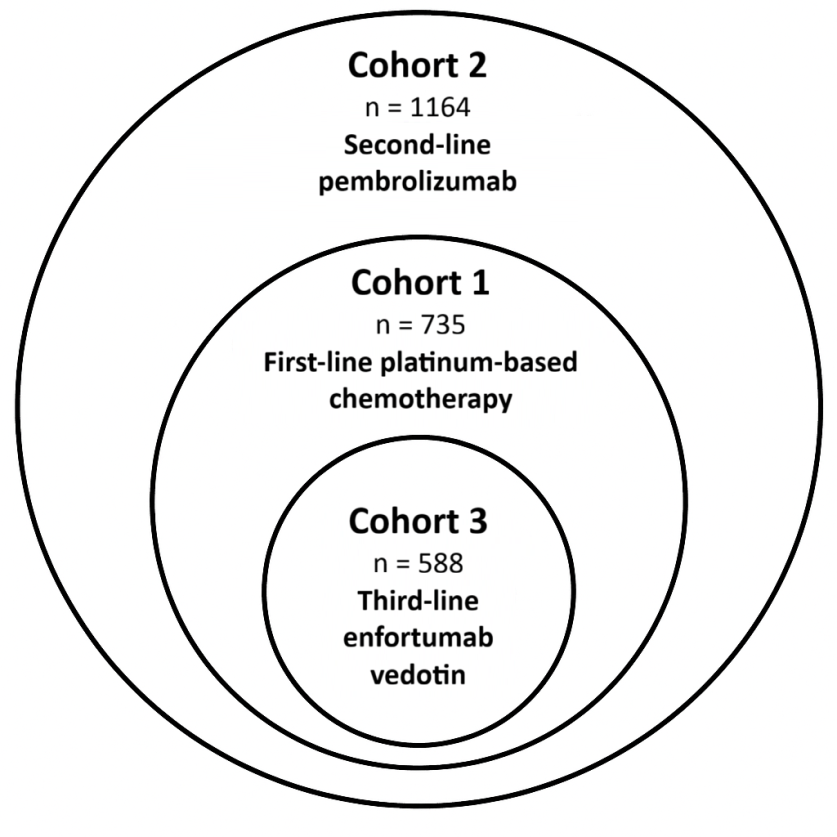
**

**Figure S3.** Hierarchical structure of the study cohorts.
